# Efficient Removal of Phenol Red Dye from Polluted Water Using Sustainable Low-Cost Sewage Sludge Activated Carbon: Adsorption and Reusability Studies

**DOI:** 10.3390/molecules29245865

**Published:** 2024-12-12

**Authors:** Salha M. Aljubiri, Ayman A. O. Younes, Eid H. Alosaimi, Mahmoud M. Abdel-Daiem, Enas T. Abdel-Salam, Walaa H. El-Shwiniy

**Affiliations:** 1 Department of Chemistry, College of Science, University of Bisha, Bisha 61922, Saudi Arabia; sragh@ub.edu.sa (S.M.A.); aayounes@ub.edu.sa (A.A.O.Y.); ealosaimi@ub.edu.sa (E.H.A.); enastaha@ub.edu.sa (E.T.A.-S.); 2 Environmental Engineering Department, Faculty of Engineering, Zagazig University, Zagazig 44519, Egypt; mmabdeldaiem@eng.zu.edu.eg; 3 Civil Engineering Department, College of Engineering, Shaqra University, Dawadmi 11911, Saudi Arabia; 4 Department of Chemistry, Faculty of Science, Suez Canal University, Ismailia 41522, Egypt

**Keywords:** adsorption, reusability, sludge, activated carbon, phenol red dye

## Abstract

The use of sewage sludge activated carbon (thickened samples ACS1 and non-thickened samples ACS2) in a variety of applications and simple environmentally friendly production techniques are attracting more and more attention. We offer here a novel environmentally friendly method based on the green synthesis of activated carbons (ACS1/ACS2) using sewage sludge (SS). These activated carbons are then used to effectively remove the water-based reactive dye phenol red (PR). The ACS1 and ACS2 produced are porous materials with an average diameter of 20.72–13.30 and 6.20–7.34 nm, respectively. These ACS1/ACS2 were analyzed using a range of characterization techniques including X-ray diffraction (XRD), Fourier transform infrared spectroscopy (FT-IR), scanning electron microscopy (SEM), transmission electron microscopy (TEM) and Brunauer–Emmett–Teller (BET) analysis. Elimination of toxic PR dye was investigated using several operational factors, including ACS1/ACS2 dose, initial PR dye concentration, pH and temperature. Under the best experimental conditions, the ACS1 and ACS2 adsorbents absorbed nearly 89.58% and 97.69% of the PR dye, respectively. It was found that both ACS1 and ACS2 adsorption corresponded to pseudo-first-order kinetics (R = 0.996 and 0.980) and fulfilled Langmuir’s (ACS1) and Freundlich’s (ACS2) models well, with maximum adsorption capacities of 65.35 and 122.72 mg/g, respectively. It was found that the adsorption processes are basically exothermic. The results suggest that sewage sludge can be effective as a low-cost and environmentally beneficial synthesis of ACS1 and ACS2 in the purification of water sources contaminated with hazardous dyes.

## 1. Introduction

Dyes in industrial wastewater are a significant environmental concern due to their toxicity and visibility even at low concentrations, which can harm aquatic ecosystems and humans. Industrial activities, especially in the textile sector [1]. Approximately 20% of global dye production is discharged into the environment by textile industries, mainly because of incomplete dye absorption and washing processes [2]. The release of untreated colored water into natural water bodies poses a threat to both aquatic life and humans because of its mutagenic and carcinogenic properties [3,4,5]. These pollutants are challenging to remove due to their stability and resistance to conventional treatment [1,6,7,8,9,10]. The elimination of dyes from water bodies has a variety of technologies including adsorption, photochemical, precipitation, advanced oxidative processes, and membrane filtration methods [4,6,8,11,12,13,14]; however, the adsorption process is considered the most used option because of its potent effectiveness, safety, low energy requirements, and affordability [1,3,5,15]. Activated carbon is the most widely used adsorbent in adsorption processes due to its high surface area and ease of use, which enable it to effectively remove both organic and inorganic contaminants from aqueous solutions [16,17,18]; moreover, it is derived from various bio-waste material that considered the most harmful residues in environment [15,16,17,18,19,20,21,22,23,24,25,26,27]. The usage of bio-waste as cheap, ecological, renewable sources can contribute to improve the sustainability of carbon material production [28]. Furthermore, it minimizes the release of greenhouse gases due to its lower sulfur and nitrogen content compared to fossil coals [20]. Sewage sludge is considered one of the most hazardous biomass wastes due to its harmful effects; moreover, it has an advantage due to its high carbon content and availability as a waste byproduct of wastewater treatment processes [22,23,24,25,26,27,29]. Utilizing sewage sludge for activated carbons production not only addresses the management of this bio-waste but also contributes to sustainability by reducing greenhouse gas emissions compared to fossil coal-based activated carbons. Previous studies have shown promising but limited adsorption capacities and surface areas for SS-derived activated carbons [22,23,24,25,26,27,29]. Thus, this study aims to improve the characteristics of the fabrication of activated carbons from thickened and unthickened sewage sludge using a novel green synthesis method. Recent studies have demonstrated that the potential of utilizing sewage sludge as a bio-waste material for producing activated carbon is promising due to its high carbon content, however, as noticed, the BET surface area was low in comparison and the same for the adsorption capacity [22,23]. Table 1 summarizes previous studies on the preparation of activated carbon from sewage sludge.

On the other hand, recent studies related to the removal of phenol red can be summarized as Mahramanlioglu et al. investigated the use of activated carbon and magnetic activated carbons (MACs) for removing phenol red from water. The results showed that activated carbon had the highest surface area of 1040 m^2^/g, while MAC12 (1:2 iron oxide-to-carbon ratio) and MAC11 (1:1 ratio) had slightly lower surface areas of 868 m^2^/g and 841 m^2^/g, respectively. The adsorption process followed the Lagergren first-order kinetic model, and equilibrium was reached in 300 min for activated carbon, 275 min for MAC12, and 240 min for MAC11. The Langmuir adsorption capacities were 149.2 mg/g for activated carbon, 120.5 mg/g for MAC12, and 111.1 mg/g for MAC11. The study also found that adsorption capacity decreased as pH increased, highlighting the impact of pH on the process [1]. Yimer et al. conducted a systematic experimental study on the kinetics and equilibrium of phenol red adsorption using teff (Eragrostis) husk-activated carbon (THAC) and husk powder (HP). The adsorption process was optimized for factors such as pH, initial dye concentration, adsorbent dosage, and contact time. They found that THAC proved to be a more effective adsorbent than HP, with maximum adsorption occurring at pH 5.2 and 5.0, respectively [5]. Iroh et al. evaluated the potential of immobilized fungal cells (*Aspergillus tubingensis* and *Aspergillus pseudonomius*) on activated carbon from rice husks for the biosorption of Congo red dye in a continuous flow-packed bed column. It assessed how various parameters—such as inlet dye concentration, feed flow rate, biosorbent dosage, and column diameter—affected the breakthrough curves of the biosorption process. The results indicated that optimal conditions for Congo red biosorption were a high biosorbent dose (4 g), a small column diameter (1.5 cm), a low initial dye concentration (50 mg/mL), and a reduced flow rate (0.5 mL/min). The maximum adsorption capacity was predicted at 66.15 mg/g. The findings suggest that the dead biomass of the studied Aspergillus species immobilized on rice husk-derived activated carbon could serve as an effective and cost-efficient biosorbent for Congo red removal in a fixed-bed column system [9]. Moreover, Mittal et al. utilized bottom ash and deoiled soya as adsorbents to remove phenol red dye from wastewater. They investigated factors such as solution pH, initial concentration, adsorbent mass, contact time, and temperature, revealing those higher temperatures increased dye uptake, indicating an endothermic adsorption process. The maximum adsorption capacity for the phenol red-bottom ash system was 2.6 × 10^−5^ mol/g at 50 °C, with the Langmuir isotherm model fitting the data well. Kinetic studies identified the adsorption mechanisms, while regeneration tests showed over 90% recovery of the adsorbents using an acidic eluent [27]. 

The novelty of this research lies in enhancing the adsorption efficiency of sewage sludge-derived activated carbons for phenol red, an anionic dye that poses environmental hazards. This study hypothesizes that optimizing the sewage sludge-derived activated carbons synthesis will yield adsorbents with superior surface characteristics and adsorption capacities, thus providing an environmentally friendly and cost-effective solution for dye removal from polluted water. From this background, the main objective of this study is to examine the adsorption of phenol red onto the activated carbon fabricated from sewage sludge with a high surface area and adsorption capacity for up taking phenol red. The adsorption of activated carbons will be investigated through the kinetics and isotherm process then analyzing the influence of the solution pH and initial concentration of phenol red, mass of activated carbon, and adsorption time on the adsorption yield, and the reusability studies.

## 2. Results and Discussion

### 2.1. Characterization of Activated Carbons ACS1 and ACS2

#### 2.1.1. FT-IR Analysis

**Figure 1:**
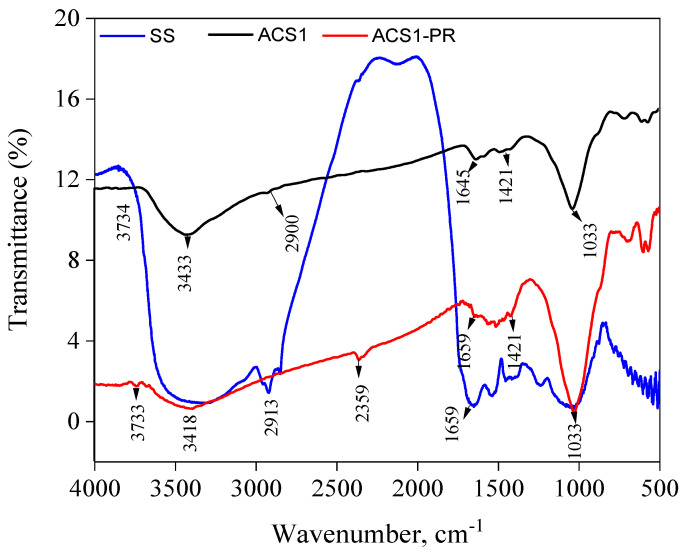
FT-IR spectra of unthicken sewage sludge (SS), Activated carbon (ACS1) and Activated carbon (ACS1-PR) after absorption phenol red.

**Figure 2:**
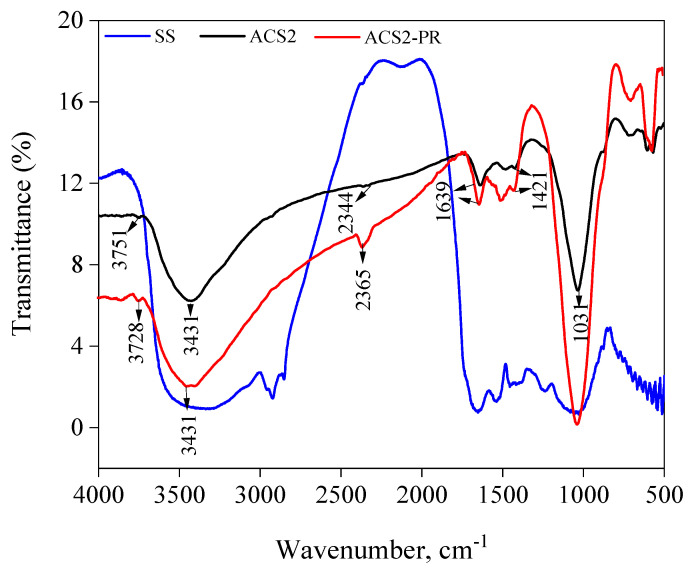
FT-IR spectra of unthicken sewage sludge (SS), Activated carbon (ACS2) and Activated carbon (ACS2-PR) after absorption PR.

The surface chemistry of activated carbon (ACS1 and ACS2) and sludge from wastewater (SS) obtained through penetration with KOH have been assessed employing FT-IR (Figure 1 and Figure 2 and Table 2). It was theorized that there are functional groups of compounds such as lactones, phenols, anhydrides, aldehydes, ketones and quinones. The acid-base composition of the activated carbon is influenced by these functional groups [30,31]. Sludge from sewage systems contains a lot of functional groups, especially from the volatile components. Si-OH compounds and physically adsorbed water molecules are associated with a broader band between 4000 and 3700 cm^−1^ [32]. Structures containing hydroxyl groups include alcohols, carboxyl structures and phenols. OH was the symbol of the carboxyl group, the key absorption band between 3800 and 2500 cm^−1^ represents it [33]. The peak at 1410 cm^−1^ is prompted by the phenolic OH groups. The degradation of the phenolic structures causes this peak to disappear at high temperatures. The peak positions of 2919 and 2850 cm^−1^ are aliphatic C–H peaks. The peak at 1036 cm^−1^ [34] indicates the C–C bond. The NH groups were the result of vibrational motions, as shown by the peak at 1541 cm^−1^. These properties lead to a structure that is rich in functional groups such as those found in sewage sludge. The activated carbon spectrum shows two remarkable peaks, 3410 and 1028 cm^−1^ respectively, indicating the presence of OH and C–O groups (Figure 1). The signal at 3383 cm^−1^ indicates the presence of hydroxyl groups in the KOH-impregnated activated carbon structure, while the peak at 1633 cm^−1^ indicates the synthesis of carbonates [35,36]. The structures of sewage sludge and activated carbon show 1410 and 1418 cm^−1^ peaks, respectively, indicating that the phenolic OH groups are still degrading. Peaks can be seen in the region of fingerprints at 605 and 440 cm^−1^ indicating the presence of mono-substituted aromatic compounds. Figure 2 displays the PR spectra and PR in combination with activated carbon (ACS1-PR, ACS2-PR). The PR spectra showed that the sulfonate group is stretched at 1480 cm^−1^. However, in the spectra of activated carbon bound to PR, this band is not visible at all. Meanwhile, a clear stretching of the OH group in PR was observed at 3293 cm^−1^. A second broadband with a core at 3429 cm^−1^ formed later when this band disappeared. This shows that PR, like other anchoring groups, can adhere to activated carbon surfaces.

#### 2.1.2. Brunauer Emmet Teller (BET) Analysis 

**Figure 3:**
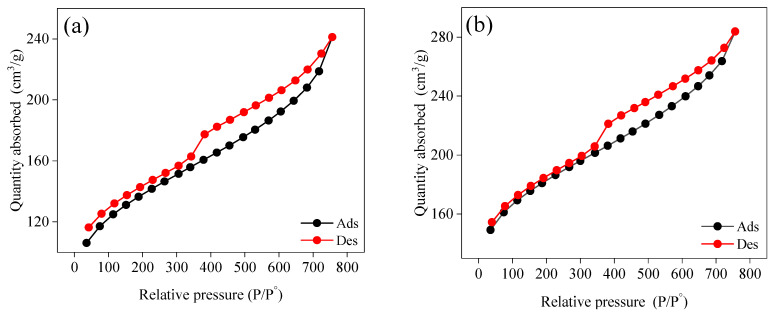
N_2_ adsorption and desorption isotherms of ACS1 (**a**) and ACS2 (**b**) samples.

The surface area of manufactured ACS1 and ACS2 was investigated using N_2_ adsorption-desorption isotherms at −196 °C (Figure 3). According to the BET classification, the N_2_ isotherms ACS1 and ACS2 exhibited a type I characteristic. This behavior is attributed to the hysteresis loop and the sharp increase at low relative pressure, as shown in Figure 3. The hysteresis loop, which is linked to capillary condensation in mesoporous solids, along with the rapid rise in micropore filling, suggests the involvement of microporous carbons with significant mesoporosity development [35,36].

According to de Boer’s categorization, both isotherms showed a B-type hysteresis loop, with the adsorption branches steep at saturation vapor pressure and the desorption branches steep at intermediate relative pressure, as would be expected given the parallel slit-shaped pores [37,38]. It appears that micropores accounted for only a small fraction of these materials, as the isotherms for ACS1 and ACS2 exhibited a mean hysteresis loop that deviated somewhat from the Type I character. The adsorption process was not yet complete at 1.0 P/P^0^, and the micropores filled considerably at lower partial pressures (<0.1 P/P^0^).

The surface areas of ACS1 and ACS2 were determined to be 336.34 m^2^/g and 498.98 m^2^/g, respectively, using the standard multi-point BET method (Figure S1a). The BJH desorption method was employed to analyze the pore size distribution of both materials. The pore sizes for ACS1 and ACS2 were measured to be 1.89 nm and 1.69 nm, respectively, as shown in Figure S1b,c. A summary of the pore size and surface area results is provided in Table 3. These results indicate that both ACS1 and ACS2 are mesoporous, which aligns with the findings from other characterization techniques, including the XRD results.

#### 2.1.3. Zeta Potential Analysis 

**Figure 4:**
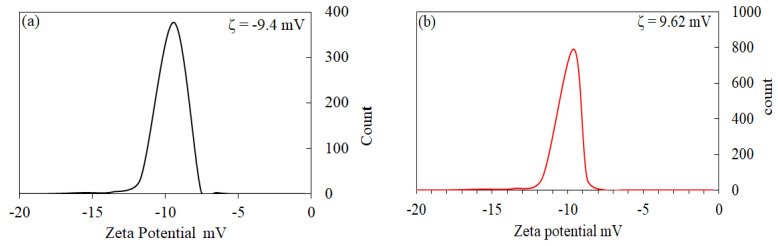
Zeta potential distribution of the ACS1 (**a**) and ACS2 (**b**) activated carbon.

The particle size distribution and surface zeta potential of ACS1 and ACS2 in an aqueous colloidal solution were determined using the Dynamic Light Scattering (DLS) technique. This method allowed for the measurement of both nanoparticle size and surface charge. The findings showed a negative zeta potential of −9.4 mV for ACS1 and −9.62 mV for ACS2, as illustrated in Figure 4a,b, indicated a strong force of repulsion between negatively charged particles and well able to adsorb positively charged pollutants.

#### 2.1.4. XRD Analysis 

**Figure 5:**
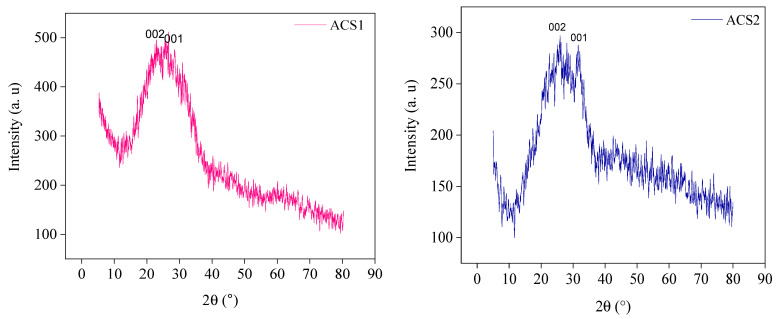
XRD spectra produced activated carbon ACS1 and ACS2.

ACS1 and ACS2 were analyzed by XRD, as shown in Figure 5. The results show that ACS1 and ACS2 have large diffraction peaks at 2*θ* = 28.48° to 30.89 and 26.08° 31.8°, respectively. These peaks are similar to the typical peaks for carbon substance (002) and graphite surface (100) [38,39]. The mean crystallite size t of the ACS1 and ACS2 was calculated to be 20.72–13.30 and 6.20–7.34 nm, respectively, as shown in Table 4. 

This calculation was performed using the standard Debye-Scherrer Equation (1):

(1) $$t=\frac{k\lambda }{\beta cos\theta }$$

where *t* is the crystallite size in nanometers (nm), *k* is a constant dependent on the crystallite shape (equal to 0.89), *λ* is the X-ray wavelength (equal to 0.1542 nm), *θ* is the diffraction angle in degrees, and *β* is the full width at half maximum intensity (FWHM) of the diffraction peak, measured in radians. The larger specific surface area and porous structure led to higher ACS2 adsorption [40]. The results showed that the activated carbons ACS1 and ACS2 had (d001) values between 0.3223 and 0.5021 nm and 0.1501–0.1778 nm, respectively (Table 4). The interlayer distances value of ACS1 are higher and ACS2 lower than the value of 0.335 nm predicted for ideal graphite [41]. A more disordered state of the investigated carbon materials could be associated with these larger distances, which could improve the ion penetration and adsorption ability of ACS1 and ACS2.

#### 2.1.5. The Ultimate Analysis

The composition of the organic matter of the SS was changed by a 2 h paralysis at 573 K (300 °C) and then by a 1 h paralysis at 1173 K (900 °C), as shown by the changes in the C, H, O and N content and the H/C, O/C and N/C ratios. After paralysis, the H/C, N/C and O/C ratios are lower than those of the raw SS. The H/C ratio drops from 0.14 (SS) to 0.04 (ACS1) and 0.06 (ACS2). The paralyzed SS appear to contain more aromatic groups than the raw SS samples due to changes in the H/C, N/C, and O/C ratios. It is possible that light organic molecules become soluble during the paralysis process [42]. The ACS2 sample had a higher H/C ratio than the other ACS1 samples, which is a distinguishing feature. This shows that the non-thickened, paralyzed sample works better in the presence of long chains (with CH_2_ groups) and more aliphatic carbon [43,44]. The higher content of aliphatic carbons contributes to the production of gaseous alkanes or light aromatic hydrocarbons. The N/C ratio can be used to quantify the degree of polymerization of organic material in sewage sludge. Higher polymerization of organic material results in fewer nitrogen-containing functional groups [44]. Consequently, a higher degree of polymerization in paralyzed sewage sludge indicates a better dewatering capacity. As shown in Table 5, ACS1 and ACS2 exhibited a much lower O/C ratio than raw SS, which means that part of the oxygen was oxidized or transferred from oxygen-containing functional groups throughout the process.

#### 2.1.6. XRF Analysis

Sewage sludge is a combination of organic material, microorganisms and minerals, mainly Si, Ca, Fe, O, K, and Cl; including nitrogen and sulfur, as well as trace metals such as Mn, Zn, and Sr. Table 6 shows an elemental screening analysis of SS, dry raw sludge sample, ACS1 and ACS2 with an X-ray fluorescence spectrometer (XRF). The water was purified and recovered at the end of the raw sewage sludge process. The metal ions that were deposited did not evaporate during heat treatment or chemical activation; instead, they remained on the carbon surface and within the pores. The metal ions produced ash, which covered the active carbon surface and clogged the pores [45]. They also restricted the surface area and prevented the use of activated carbon. Si, Ca, and Fe ions took up most of the raw sewage sludge. There were no obvious differences in metal content between ACS1 and ACS2. Matrices ACS1 and ACS2 contained larger total amounts of Fe, Cu, Zn, Mn, and Ca than the raw sewage sludge. This could be due to the accumulation of heavy metals in the ACS1 and ACS2 matrix during pyrolysis, which leads to a lower weight loss of the metals than of the organic molecules [46]. The heavy metal concentrations in the sludge, ACS1 and ACS2 followed a trend of Ca > Si > Fe > P > K > Cl > Ti > Zn.

#### 2.1.7. Morphology of Activated Carbon

**Figure 6:**
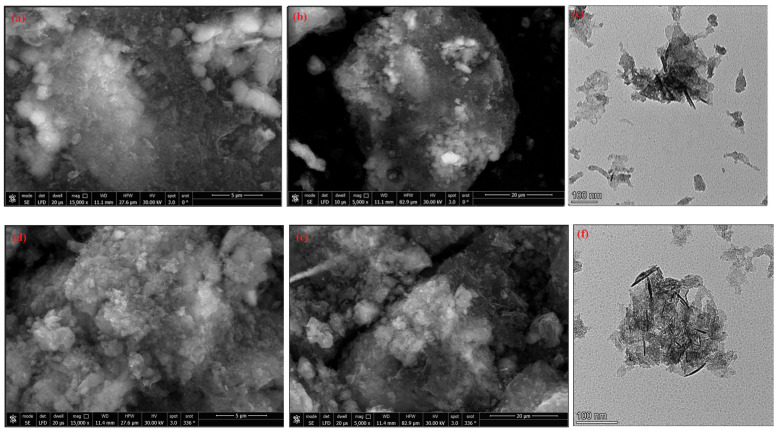
SEM micrographs of ACS1 (**a**,**b**), ACS2 (**d**,**e**) and TEM image ACS1 (**c**) and ACS2 (**f**).

Figure 6 shows the physical morphology of ACS1 and ACS2. The graph shows a significant change in morphology, especially in size and pores. The SEM images of ACS1 (Figure 6a,b) and ACS2 (Figure 6d,e) show broad outer surfaces with considerable porosity. This is particularly successful in the removal of pollutants from water by adsorption techniques. In the high-resolution TEM image of ACS1 (Figure 6c) and ACS2 (Figure 6f), the disordered and porous structure of the carbon flakes can be seen. The distribution of particle sizes is 20.72–13.30 nm for ACS1 and 6.20–7.34 nm for ACS2. This is consistent with the results of the XRD investigation. The surface area of ACS1 and ACS2 is increased by these irregularly sized pores, which also leads to an improvement in efficiency and adsorption capacity.

### 2.2. Adsorption Study

#### 2.2.1. Influence of the ACS1 and ACS2 Dosage

**Figure 7:**
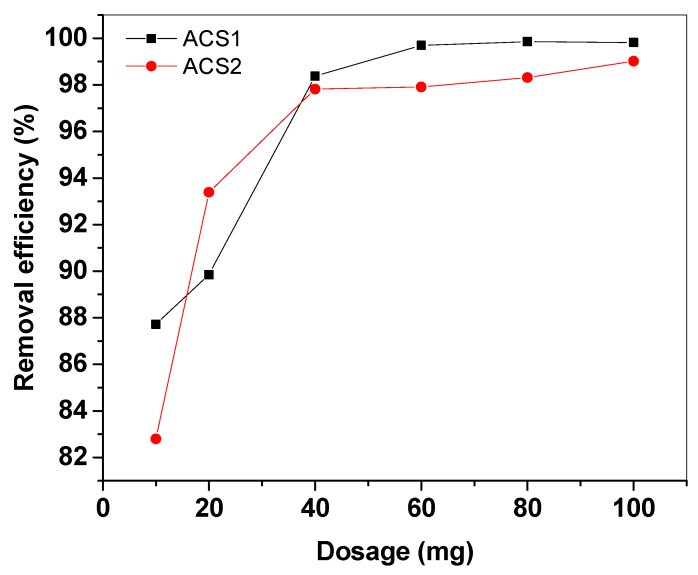
Effect of dose of adsorbent ACS1 and ACS2 on the removal of PR dye at 30 °C, initial concentration at 100 mg and pH = 7.

To investigate the change in adsorption as a function of the amount of adsorbent, different doses of ACS1 and ACS2 from 0.01 g to 0.1 g were added to 25 mL volumetric flasks containing 0.1 g of the PR dye and pH = 7. The study was carried out at 30 °C. Increasing the mass of ACS1 and ACS2 from 0.01 g to 0.1 g resulted in an increase in the adsorbed percentage from 87% and 82.8% to 99.8% and 99.01% for PR dye, respectively. It was observed that the adsorption increased with increasing amount of adsorbent (Figure 7). This trend is largely related to the fact that as the amount of adsorbent increases, the adsorptive surface area also increases, providing a greater number of active sites for adsorption [47].

#### 2.2.2. Influence of Initial Dye Concentration

**Figure 8:**
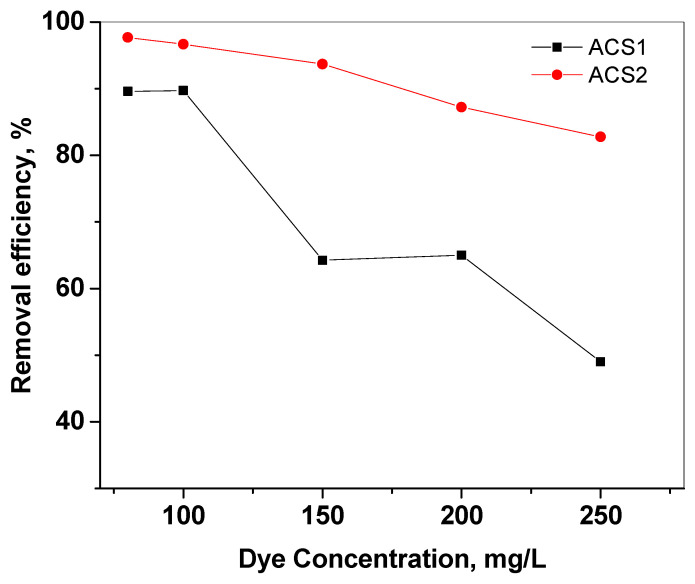
The color removal efficiency of ACS1 and ACS2 for different initial concentrations of PR dye at 30 °C adsorbent dose = 40 mg, pH 7.0.

The effect of initial dye concentration on dye removal efficiency was investigated in a wide range of PR dye concentrations (80 to 250 mg/L), keeping the optimal dose of ACS1 and ACS2 adsorbents (40 mg) and the solution volume at 25 mL. Adsorption at 30 °C decreased for both ACS1 and ACS2 as the dye concentration increased (Figure 8). The removal efficiency for 80 mg PR is 89.5% and 97.6% with ACS1 and ACS2, respectively, but for 250 mg PR, the removal efficiency is 49.0% and 82.77% with ACS1 and ACS2, respectively. As the concentration of the dye solution increases, the adsorption sites become saturated and prevent further adsorption of the PR dye molecules at all temperatures [48].

#### 2.2.3. Influence of pH

**Figure 9:**
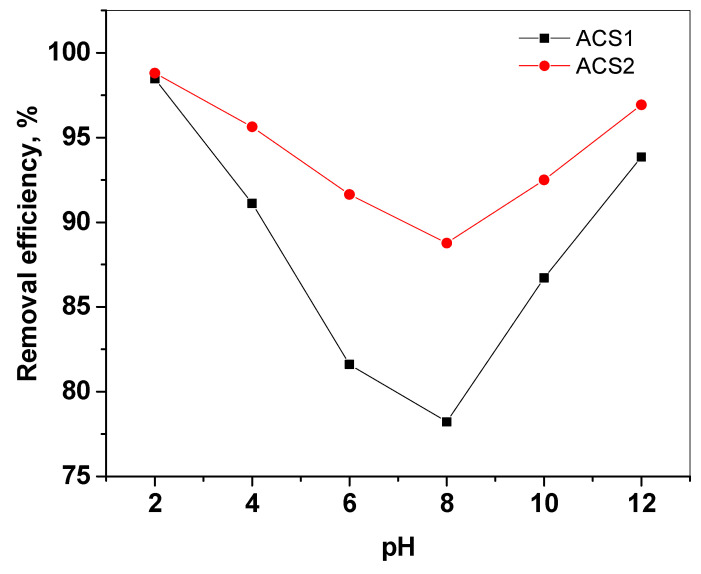
Effect of pH on the removal efficiency of PR dye by ACS1 and ACS2 at 30 °C, initial concentration; 100 mg, adsorbent dose; 40 mg.

One of the most important variables in PR dye adsorption is pH, which directly influences the dissociative and adsorptive activity of the PR dye on the ACS1 and ACS2 adsorbent surfaces. Therefore, the effect of pH on PR removal efficiency was investigated using (0.1 N) NaOH or (0.1 N) HCl solutions in the pH range (2–12) under all other similar conditions, including ACS1 and ACS2 dose, initial dye concentration, and contact time. Figure 9 shows that as pH increases, adsorption decreases up to pH 8, but as pH increases (up to pH 12), both ACS1 and ACS2 adsorbents absorb more. The initial decrease in the amount adsorbed with increasing pH is due to increased protonation by neutralization of negative charges on the adsorbent surface [1]. For the following studies, a pH value of 7 was chosen as it provides optimum adsorption capacity under neutral conditions.

#### 2.2.4. Influence of Temperature

The temperature influences the solubility of the dye, the swelling of the adsorbent and the exothermic or endothermic adsorption equilibrium [49]. Figure S2 shows the impact of temperature on color elimination efficiency for PR dye at temperatures between 30 °C and 60 °C while maintaining all optimal parameters. As can be seen from the figure, the PR dye studied at room temperature (30 °C) using ACS1 and ACS2 achieved a maximum color removal efficiency of 89.7% and 93.46%, respectively. In addition, increasing the temperature above room temperature reduced the efficiency of color removal. As the process temperature increased, the removal efficiency decreased, suggesting that ACS1 and ACS2 remove the PR dye in an exothermic process [50].

**Figure 10:**
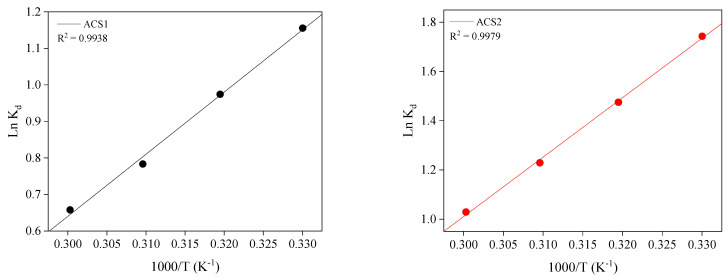
Van’t Hoff plot for the adsorption of phenol red ACS1 and ACS2 at different temperatures.

To examine how temperature affects PR absorption by ACS1 and ACS2, the experiments were performed at four temperatures (Figure 10). As shown in Table 7, the ΔH^0^ value of ACS1 and ACS2 was −141.4594 and −200.6434 J mol^−1^, indicating an exothermic reaction; thus, the adsorption capacity of ACS1 and ACS2 was inhibited under the conditions of high reaction temperature. A negative ΔS^0^ value of ACS1 and ACS2 indicated an improved ordering of the solid interface during the adsorption of PR [51]. According to previous studies [52,53], a Gibbs free energy (ΔG^0^) value between −20 and 0 kJ mol^−1^ indicates a physical adsorption process, while a value between −400 and −80 kJ mol^−1^ suggests chemical adsorption. The ΔG^0^ values for ACS1 and ACS2 were found to be between −20 and 0 kJ mol^−1^ at various temperatures, indicating that the adsorption process is physical, driven by Vander Waals forces or weak electrostatic interactions. Additionally, the absolute ΔG^0^ values for ACS1 and ACS2 increased with rising reaction temperature from 303 to 353 K. This further confirms that higher temperatures enhance the adsorption capacities of ACS1 and ACS2 for PR.

The value of Gibbs free energy (ΔG^0^) between −20 and 0 kJ mol^−1^ indicates a physical adsorption process and the value of chemical adsorption was between −400 and 80 kJ mol^−1^ [52]. 

ACS1 and ACS2 have ΔG^0^ values ranging from −20 to 0 kJ mol^−1^ at different temperatures, indicating physical adsorption by Vander Waals forces or slight electrostatic contact. The absolute ΔG^0^ values of ACS1 and ACS2 increased progressively as the reaction temperature increased from 303 to 353 K. This study shows that higher temperatures increase the adsorption capacity of ACS1 and ACS2 for PR.

### 2.3. Kinetics Dynamics of PR on ACS1 and ACS2

**Figure 11:**
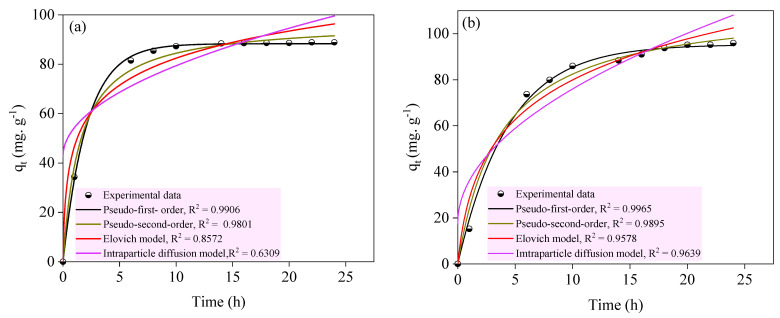
Fitting results of adsorption kinetic model for adsorption of PR onto ACS1 (**a**) and ACS2 (**b**).

At different initial dye concentrations (100–300 mg/L), the time-dependent adsorption profiles for the removal of PR dye by the selected activated carbons (ACS1 and ACS2) were determined together with the corresponding constants: 6.2–6.6 pH of the solution, 10 mL volume of PR, 25 ± 2 °C operating temperature, 10 mg of the adsorbent, and 0.0–24 h contact time. As shown in Figure 11 and Table 8, pseudo-first order equations, pseudo-second order equations, Elovich equations, and intraparticle diffusion equations were used to describe the data obtained to enable appropriate analysis. Table 8 shows that the measured equilibrium adsorption capacity (q_e,exp_) of ACS1 was 88.3474 mg g^−1^, while that of ACS2 increased to 95.3360 mg g^−1^. The correlation coefficient (R^2^ = 0.9906) of the pseudo-first-order kinetics of ACS1 was higher than that (R^2^ = 0.9801) of the pseudo-second-order kinetics, and the adjusted q_e_ value (88.9329 mg g^−1^) of the first-order kinetics was closer to the q_e,exp_ value (88.3474 mg g^−1^). This finding indicates that the pseudo-first-order kinetic model, which is primarily determined by the diffusion process, is better suited to explain the adsorption process of PR from ACS1. Similarly, the PR adsorption process of ACS2 was in better agreement with the pseudo-first-order model, which was mainly determined by the diffusion process. Notably, the pseudo-first-order model of ACS2 outperformed ACS1 in goodness of fit (R^2^ = 0.9962), indicating that the diffusion process plays a significant role in the PR adsorption process of ACS2. In this case, the intraparticle diffusion model provided additional evidence supporting the role of the diffusion process [53]. Table 9 shows that the intraparticle diffusion model of ACS2 has a correlation coefficient (R^2^) of 0.9658 and that the adsorption equilibrium time of ACS2 is more than 15 h. These results indicate the significant contribution of the intraparticle diffusion process. The Vander Waals force, which is considered a component of weak molecular interaction, ultimately accelerated this physical diffusion [54]. The R^2^ value of the ACS1 intraparticle diffusion model was 0.6305, indicating that the interfacial diffusion process is the most important controlled step in ACS1.

### 2.4. Adsorption Isotherm Analysis

**Figure 12:**
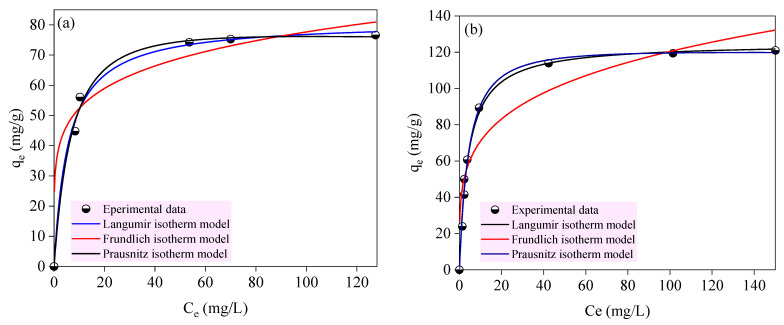
Adsorption isotherms of PR on both activated carbons ACS1 (**a**) and ACS2 (**b**).

The adsorption isotherms of PR onto ACS1 and ACS2 are shown in Figure 12. The adsorption isotherms onto both carbons have L-behavior [55]. A concave curve, as depicted in Figure 12, is formed when the ratio of the pollutants adsorbed onto the carbon material to their residual concentration in the aqueous solution decreases. The experimental data were analyzed using the Langmuir, Freundlich, and Prausnitz-Radke isotherm models for prediction. The adsorption capacity of ACS2 is greater than ACS1 that may be attributed to the high surface area of ACS2 (524.353 m^2^/g) than that of ACS1 (403.607 m^2^/g) (Table 9). The Prausnitz–Radke model (%D = 14.34) was the best at describing the adsorption isotherm of PR onto ACS1, closely followed by the Freundlich model (%D = 14.95), and then the Langmuir model (%D = 16.18) that means the adsorption process on ACS1 is near to be a multilayer adsorption [16,28]. However, the adsorption of phenol red onto ACS2 was descripted by Langmuir model (%D = 13.44) and was fitted slightly better than the Prausnitz–Radke model (%D = 13.53) and followed by the Freundlich model (%D = 21.53). This indicates that the contribution of monolayer adsorption on ACS2 was greater than that of the multilayer adsorption process [56,57,58]. As shown in Table 10, the adsorption affinity (B) was higher than 1.00 in the case of ACS1 which means the adsorption process is unfavorable, however, it was lower than 1.00 in the case of ACS2 which means the adsorption process is favorable [59], these findings confirmed by the adsorption capacity of ACS2 was higher than ACS1 (Table 9). The applied Freundlich isotherm model toexperiment data showed that, for both activated carbons the sorption intensity or the adsorbate binds more readily to the adsorbent at lower concentrations. This often corresponds to a heterogeneous surface with sites of varying affinity [60].

### 2.5. Reusability of ACS1 and ACS1

**Figure 13:**
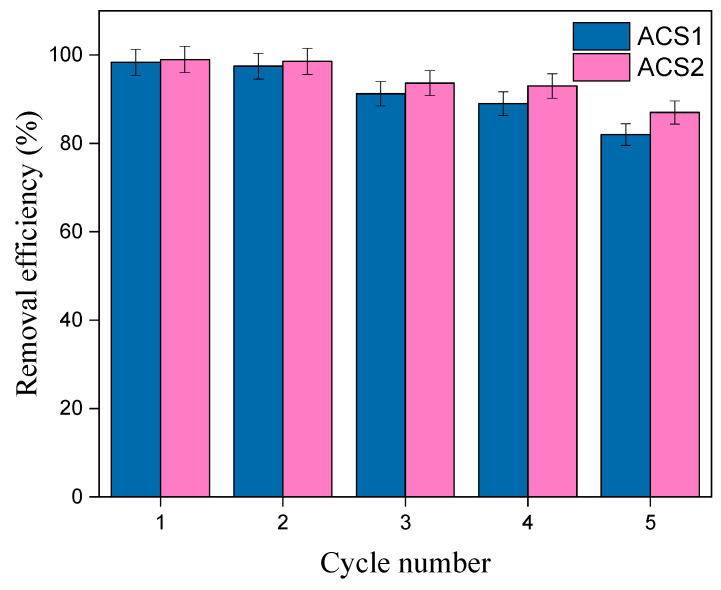
Reusability of ACS1 and ACS2 adsorbents.

The ability of sorbents to undergo several regeneration cycles is of great economic importance due to their influence on production costs. The PR loaded with ACS1 and ACS2 was shaken in ethyl alcohol for five hours, rinsed with ethyl alcohol and heated at 50 °C for five hours (heat desorption in a steamed oven), and the recovery of the adsorbents ACS1 and ACS2 was examined. As shown in Figure 13, ACS1 and ACS2 adsorbents exhibited persistently high affinity and maintained efficient PR removal of over 82.12% and 87.00% for ACS1 and ACS2, respectively, even after five consecutive regeneration cycles. These results highlight the efficacy and recyclability of synthetic ACS1 and ACS2 as PR adsorbents and emphasize their economic viability for repeated use.

### 2.6. Comparison of the Adsorption of Phenol Red with Other Adsorbents

Table 10 compares the maximum adsorption capacity measured with the Langmuir isotherm for PR adsorption on ACS1 and ACS2 and on different adsorbents. In contrast to previous studies, the adsorbents ACS1 and ACS2 have a strong adsorption affinity for the PR dye. The enormous surface area of ACS1 and ACS2, which serve as adsorption sites for the PR dye, could explain these results. The comparison shows that ACS1 and ACS2 are promising adsorbents for the removal of PR dye from the water ecosystem.

## 3. Experimental

### 3.1. Chemicals

In the studies carried out, the reagents and chemicals used were of analytical grade and were employed without any additional purification. The following chemicals were obtained from their respective suppliers and used as is in the experimental process: Sigma-Aldrich (St. Louis, MO, USA) supplied the phenol red dye (PR, C_19_H_14_O_5_S). In addition, hydrochloric acid (HCl, 37%) and sodium hydroxide (NaOH, 99.95%) were supplied by Sigma-Aldrich. Deionized water was used to prepare each solution.

### 3.2. Activated Carbon Production

**Figure 14:**
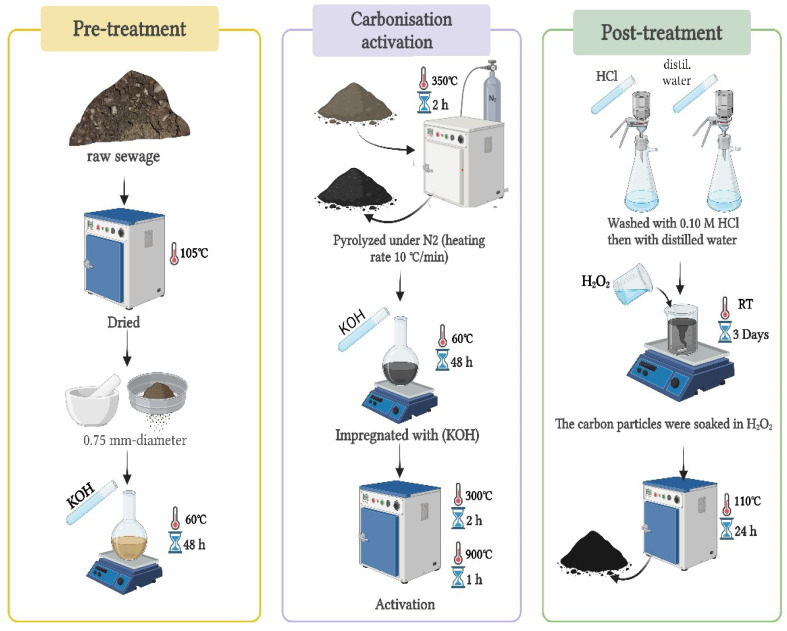
The synthesis process of activated carbon (ACS1/ACS2).

The two forms of raw sludge used to produce activated carbons are thickened samples (ACS1) and un-thickened samples (ACS2). In this process, raw sewage sludge is removed from a dry bed at a wastewater treatment plant in Bisha, in the Asir region of southwest Saudi Arabia. Figure 14 depicts the synthesis of activated carbon ACS1 and ACS2. 

### 3.3. Measurements

The morphology of ACS1 and ACS2 was performed with the field emission microscope (FE-SEM, JEOL JSM-6500F, Akishima, Japan) and the transmission electron microscope (TEM, JEM 2100, 200 Kv). The nitrogen absorption-desorption test was performed with the BET device (Quantachrome, Boynton Beach, FL, USA). The XRD pattern was recorded using model ADX2500 (Angstrom Advanced Inc., Stoughton, MA, USA) at 2θ = 0.5°–60° and CuK_α_ of 1.5406 nm. The phenol red content was determined using the T80 UV/Vis double spectrometer (PG Instruments Ltd., Lutterworth, UK) at λ_max_ 430 nm. The FT-IR 460 PLUS spectrophotometer (Thermo Scientific Nicolet^TM^ iS10, Waltham, MA, USA) was used to record the IR spectrum in KBr discs in the 4000–400 cm^−1^ range. Adwa pH metre (model AD 1030, Danang, Romania). Digital hot plate stirrer (model MSH-20D, Republic of Korea). PLC series centrifuge (model PLC-03, Portsmouth, OH, USA) with a power of 220 V/50 HZ; 0.65 A. CHNS analysis was measured using an elemental analyzer (Elementar, Langenselbold, Germany). The zeta potential was determined using a Zetasizer Nano series device (Nano ZS, Malvern, UK).

### 3.4. Preparation of Phenol Red Dye Solutions

The stock solution of phenol red [66] 100 ppm (PR, MW: 354.38, Table 11) was prepared by dissolving the appropriate amount of phenol red dye in water and adding deionized water to reach the 100 mL limit. Different amounts of phenol red between 5 and 40 ppm were prepared by dilution. All adsorption measurements were performed at room temperature. The straight line of the calibration curve for determining the concentration of phenol red was obtained with R^2^ = 9947. Figure S3 shows a calibration curve for determining the concentration of phenol red. Experimental molar absorption coefficient of acidic phenol red is 11,360 L·mol^−1^·cm^−1^ at 430 nm. Most of the literature calculated the molar absorption coefficient for basic phenol red at 560 nm [67].

### 3.5. Adsorption Batch 

#### 3.5.1. Procedures of Decolorizing Dye 

The adsorption properties were determined by primary analysis. To investigate the influence of critical factors such as concentration, amount of adsorbent and pH, batch studies were performed with 25 mL of PR dye solution at room temperature. The adsorption tests were carried out at temperatures from 30 °C to 80 °C for the temperature experiment. To study the kinetics of adsorption, samples were taken from the incubated dye solution at regular intervals (1, 2, 3, 4, 5, 7, 9, 13, 15, 22, 24 h). When NaOH (0.1 M) and HCl (0.1 M) solutions were used, the initial pH influence on the adsorption behavior was between 2 and 12. All experiments were performed three times. ACS1 and ACS2 (40 mg) were selected as adsorbents and were shaken at 140 rpm for one hour on water bath shaker. Then, the solutions were centrifuged at 6000 rpm for 10 min. The amount of adsorbed dye was measured using a UV-Vis spectrophotometer and calibration curves at λ_max_ = 430 nm. The removal efficiency (% *R_e_*) and the adsorption capacity (*Q_e_*, mg·g^−1^) of PR were calculated from the following Equations (2) and (3):

(2) $$\%R_{e}=\frac{C_{0}-C_{e}}{C_{0}}\times 100$$

(3) $$Q_{e}=\frac{C_{0}-C_{e}}{m}\times V_{0}$$

where C_0_ and C_e_ are the initial and equilibrium concentrations of PR, mg/·L; V_0_ is the volume of PR solution, L; m is the adsorbent dosage weight, g.

#### 3.5.2. Adsorption Isotherms

The adsorption isotherms of the two activated carbons ACS1 and ACS2 were calculated using initial concentrations of phenol red between 10 and 250 mg/L. The adsorption isotherm models of Langmuir, the experimental data were analyzed using the Freundlich and Prausnitz–Radke Equations (4)–(6). The adsorption process and the definition of the adsorption mechanism and type are simplified by these models, which are the most used isothermal adsorption models:

(4) $$X_{\mathrm{eq}}=\frac{BX_{m}C_{e}}{1+BC_{e}}$$

(5) Xeq=KFCe1nF

(6) $$q=\frac{aC_{A}}{1+b{C_{A}}^{\beta }}$$

where X_eq_ is the adsorption yield (mg/g), X_m_ is the adsorption capacity (mg/g), B is the Langmuir yield constant (L/mg), C_e_ is the equilibrium concentration of the contaminant (mg/L), 1/n_F_ is the heterogeneity of the AC surface, K_F_ is the relative adsorption capacity, and a (L/g), b (L^β^/mg^β^) and β are the constants [16,17,18].

Equation (7) is applied to calculate the average absolute percentage deviations for all adsorption isotherm models:

(7) $$\%D=\frac{1}{N}\sum_{i=1}^{N}\left|\frac{X_{\exp}-X_{\mathrm{pred}}}{X_{\exp}}\right|\times \;100\%$$

where %D represents the deviation percentage, N denotes the experiment number, X_exp_ is the experimental adsorption yield in mg/g, and X_Pred_ is the predicted adsorption yield in mg/g.

#### 3.5.3. Adsorption Kinetics 

Pseudo-first order (PFO) and pseudo-second-order (PSO) models were fitted to the experimental data to study the adsorption kinetics of PR on ACS1 and ACS2. The mathematical models (8, 9) related to these data can be represented in this way:

(8) $$\ln\left(q_{e}-q_{t}\right)=lnq_{e}-k_{1}t$$

(9) $$\frac{t}{q_{t}}=\frac{1}{k_{2}q_{e}^{2}}+\frac{t}{q_{e}}$$

where: q: Weight of adsorbed adsorbate, mg/g, q_pred,1_: Weight of adsorbed adsorbate expected from the 1st order kinetic model, mg/g, q_pred,2_: Weight of adsorbed adsorbate expected from the 2nd order kinetic model, mg/g, k_1_: The 1st order kinetic model rate constant, 1/min, k_2_: The 2nd order kinetic model rate constant, g/mg/min, and t: Time, min.

#### 3.5.4. Statistical Analysis

A one-way ANOVA (SPSS 20.0, IBM, Armonk, NY, USA) was used to analyze the adsorption capacity (Q_e_) and elimination efficiency (R_e_) data with a significance level of *p* < 0.05.

## 4. Conclusions

Activated carbon was successfully synthesized using sewage sludge, demonstrating a simple, cost-effective and environmentally friendly method to produce ACS1/ACS2. This study focuses on the successful application of nanotechnology in water purification. The results of XRD, IR, CHNS, SEM, TEM, XRF, and BET illustrate the effective preparation of ACS1/ACS2 as well as the broad outer surfaces with significant porosity. The synthesized ACS1/ACS2 has a remarkable ability to adsorb phenol red (PR) dye. The ACS1/ACS2 absorbed about 89.58% and 97.69% of PR dye, respectively. The results show that the pseudo-first-order kinetic model describes the adsorption. The adsorption of the PR dye was exothermic and spontaneous, according to the thermodynamic parameters of the process. We conclude that the use of PR dye to decolorize water is feasible with the simple, efficient, and cost-effective adsorption approach.

## Figures and Tables

**Table 1:** Surface area of some prepared sewage sludge activated carbon and its adsorption yield.

Chemical Agent	Contaminant	BET Surface Area (m^2^/g)	Adsorption Capacity (mg/g)	Ref.
KOH	Cu^2+^ and Cd^2+^	173 to 190	16 and 17.6	[22]
KHCO_3_	I^−^ and Methylene blue	952	882 and 162	[23]
KOH	--	378	--	[29]
ZnCl_2_	Phenol and CCl_4_	647	47 and 8	[24]
ZnCl_2_ and H_3_PO_4_	Cu^+2^	377 and 291	11 and 8	[25]
Pyrolusite addition	Cu^2+^ Pb^2+^ and Cd^2+^	328 to 365	52, 65 and 7	[26]
ZnCl_2_	Cu^2+^, Pb^2+^, Cd^2+^ and Ni^2+^	1094	238, 96, 88 and 52	[27]

BET: Brunauer, Emmett, and Teller.

**Table 2:** Possible assignments of different wavenumbers for SS, ACS1, ACS1-PR, ACS2 and ACS2-PR.

Possible Assignments		Band Position (cm^−1^)			
Infrared Spectra (cm^−1^)	SS	ACS1	ACS1-PR	ACS2	ACS2-PR
OH stretching in free alcohol	3697	3734	3733	3751	3728
C=O stretching (amide I) O=C-NHR	2913	2365	2359	--	2352
O–H stretching within intermolecular bonding	3431	3433	3418	3438	3418
C=C (trans)	1659	1645	1645	1645	1645
C=C (cis)	1403	1421	1421	1421	1421
C–N stretch of aliphatic amine groups	1060	1021	1021	1021	1021
C–O stretching	1035	1031	1033	1031	1031

**Table 3:** Surface area, pore volume, and pore size distribution of ACS1 and ACS2 by BET analysis.

Material	Surface Area (m^2^/g)			Pore Volume		Por Size (nm)	
	Single Point BET m^2^/g	Multipoint BET m^2^/g	DFT Method Cumulative Surface Area m^2^/g	BJH Adsorption Cumulative Volume of Pore (m^2^/g)	BJH Desorption Cumulative Volume of Pore (m^2^/g)	BJH Adsorption Average Pore Diameter (4V/A)	BJH Desorption Average Pore Diameter (4V/A)
ACS1	342.2	336.34	408.73	0.1439	0.1470	1.89	1.90
ACS2	498.386	498.98	682.05	0.1735	0.185	1.688	1.90

**Table 4:** Microcrystalline structure of ACS1 and ACS2 samples.

Samples	2θd_100_ (^o^)	FWHM (β^o^)	hkl	*d*_100_ (nm)	$L_{c}^{100}(nm)$	*t* (nm)
ACS1	28.48	0.2542	002	0.5021	0.4122	20.7265
	30.89	0.1641	100	0.3223		13.3042
ACS2	26.08	0.5428	002	0.1778	0.1639	7.3394
	31.8	0.4644	100	0.1504		6.2083

**Table 5:** Ultimate analysis of sewage sludge samples.

Samples	Yield %	C (wt%) ^a^	H (wt%) ^a^	O (wt%) ^b^	N (wt%) ^a^	H/C	O/C	N/C	Ash %
Raw-SS		66.7	9.2	14.3	9.3	0.14	0.21	0.14	13.9
ACS1	86.9	87.2	3.3	2.1	2.1	0.04	0.02	0.02	8.60
ACS2	89.4	90.7	5.7	3.6	1.3	0.06	0.04	0.01	1.60

^a^ = Determined by elemental analysis; ^b^ = calculated from the difference of mass conservation law.

**Table 6:** Summarized XRF analysis of the ash of activated carbon produced from raw sewage sludge.

Samples	XRF Analysis Results (wt%)									
	Ca	Cl	Fe	K	P	Si	Ti	Sr	Zn	Mn
SS	11.21	1.01	8.02	1.03	4.08	13.20	1.34	0.12	0.35	0.14
ACS1	20.54	6.01	10.02	4.36	3.98	13.74	1.57	0.32	1.22	0.33
ACS2	21.89	2.56	13.74	2.45	4.60	12.69	2.00	0.45	1.03	0.28

**Table 7:** Thermodynamic Parameters of ACS1 and ACS2.

Sample	T/K	ΔG^0^ (kJ mol^−1^)	ΔH^0^ (J mol^−1^)	ΔS^0^ (J K^−1^·mol^−1^)
ACS1	303	−10.8753	−141.4593	−37.1178
	313	−11.4323		
	323	−11.8034		
	353	12.1746		
ACS2	303	−15.6922	−200.6434	−51.7904
	313	−16.2104		
	323	−16.7283		
	333	−17.2462		

**Table 8:** Kinetic Parameter for PR dye adsorption on ACS1 and ACS2 adsorbate.

Kinetic Models	Parameters	ACS1	ACS2
Pseudo-first-order	q_e,exp_ (mg·g^−1^)	88.3474	95.3360
	q_e,cal_ (mg·g^−1^)	88.9329	94.9306
	k_1_ (h^−1^)	0.6378	0.2264
	R^2^	0.9906	0.9962
Pseudo-second-order	q_e,exp_ (mg·g^−1^)	97.3748	113.4372
	q_e,cal_ (mg·g^−1^)	91.1123	98.1064
	k_2_ (g·mg^−1^·h^−1^)	0.0067	0.0024
	R^2^	0.9801	0.9883
Elovich model	α	280.1444	50.4646
	β	0.0628	0.0374
	R^2^	0.8572	0.9577
Intraparticle diffusion	C_i_ (mg·g^−1^)	42.6507	17.5995
	k_id_ (g·mg^−1^·h^−0.5^)	11.6172	18.4712
	R^2^	0.6305	0.9658

**Table 9:** Adsorption parameters of PR on ACS1 and ACS2 were calculated using the Langmuir, Freundlich, and Prausnitz-Radke adsorption isotherm models.

Carbon	Langmuir				Freundlich			Prausnitz–Radke			
	X_m_^(a)^ (mg/g)	B ^(b)^ (L/mg)	BX_m_^(c)^ (L/g)	%D	K_F_^(d)^ (L/g)	1/n_F_^(e)^	%D	a ^(f)^ (L/g)	b ^(g)^ (L/mg)	β ^(h)^	%D
ACS1	65.35	4.8	313.68	16.18	42.35	0.12	14.95	773.36	16.01	0.91	14.34
ACS2	122.72	0.29	35.59	13.44	37.85	0.30	21.53	37.01	0.32	0.99	13.53

Note: ^(a)^ X_m_: Adsorption capacity (mg/g); ^(b)^ B: adsorption affinity (L/mg); ^(c)^ BX_m_: adsorbent–adsorbate relative affinity (L/g); ^(d)^ K_F_: relative capacity for adsorption (L/g); ^(e)^ 1/n_F_: sorption intensity or surface heterogeneity; ^(f)^ a: constant (L/g); ^(g)^ b: constant (L/mg); and ^(h)^ β: constant.

**Table 10:** Comparison of isotherm constants for the phenol adsorption onto adsorbents.

Adsorbent	Langmuir Constant			
	q_m_ (mg/g)	K_L_ (L/mg)	R^2^	Reference
Sawdust	0.0221	10.12	0.911	[61]
Granular activated carbon	49.7	0.109	0.994	[62]
Bagasse fly ash	23.830	0.088	0.989	[63]
Bentonite	0.842	0.0158	0.997	[64]
Activated red mud	1.580	0.18	0.876	[65]
ACS1	65.35	4.8	0.980	This work
ACS2	122.72	0.29	0.988	This work

**Table 11:** Characteristics and chemical structure of phenol red dye (PR).

	Phenol Red Dye (PR)
Dye IUPAC name	3,3-Bis(4-hydroxyphenyl)-3*H*-benzo[c][1,2]oxathiole 1,1-dioxide
Chemical structure [68]	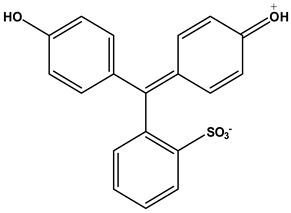
Molecular formula	C_19_H_14_O_5_S
Chemical class	Phenolsulfonphthalein
λ_max_	430 nm
Type	anionic dye
Solubility	Soluble in NaOH, sparingly soluble in water (8.3 g/100 mL at 20 °C), but soluble in most organic solvents, such as ethanol, methanol, diethyl ether, acetone, chloroform.
CAS Number	Phenol red 143-74-8

## Data Availability

The datasets used and/or analyzed during the current study are available from the corresponding author upon reasonable request.

## Supplementary Materials

The following supporting information can be downloaded at: https://www.mdpi.com/article/10.3390/molecules29245865/s1, Figure S1: Surface area plot for (a) ACS1 and for ASC2, (b) BJH desorption P^0^ distribution for ACS1 and for ACS2, and (c) differential pore volume plot for ACS1 and ACS2; Figure S2: The impact of temperature variations on PR dye removal effectiveness using ACS1 and ACS2 at initial concentration; 100 mg, adsorbent dose; 40 mg and pH = 7; Figure S3: Calibration curve for determining the concentration of phenol red.
