# Supplementary material for: Efficient Removal of Phenol Red Dye from Polluted Water Using Sustainable Low-Cost Sewage Sludge Activated Carbon: Adsorption and Reusability Studies
Source: Molecules. 2024 Dec 12;29(24):5865. doi: 10.3390/molecules29245865 (PMC11677155; doi:10.3390/molecules29245865)
Supplement: Supplementary file 1 [file molecules-29-05865-s001.zip › molecules-3307763-supplementary.pdf]

Supplementary Materials

Efficient Removal of Phenol Red Dye from Polluted Water Using Sustainable Low-Cost Sewage Sludge Activated Carbon: Adsorption and Reusability Studies

Salha M. Aljubiri [1], Ayman A. O. Younes [1], Eid H. Alosaimi [1], Mahmoud M. Abdel-Daiem [2,3], Enas T. Abdel-Salam [1,4] and Walaa H. El-Shwiniy [1*]

1. Department of Chemistry, College of Science, University of Bisha, Bisha, 61922, Saudi Arabia.
2. Environmental Engineering Department, Faculty of Engineering, Zagazig University, Zagazig 44519, Egypt.
3. Civil Engineering Department, College of Engineering, Shaqra University, Al-Duwadmi 11911, Saudi Arabia.
4. Department of Chemistry, Faculty of Science, Suez Canal University, Ismailia 41522, Egypt.

* Correspondence: whelmy@ub.edu.sa (W.H.E.).

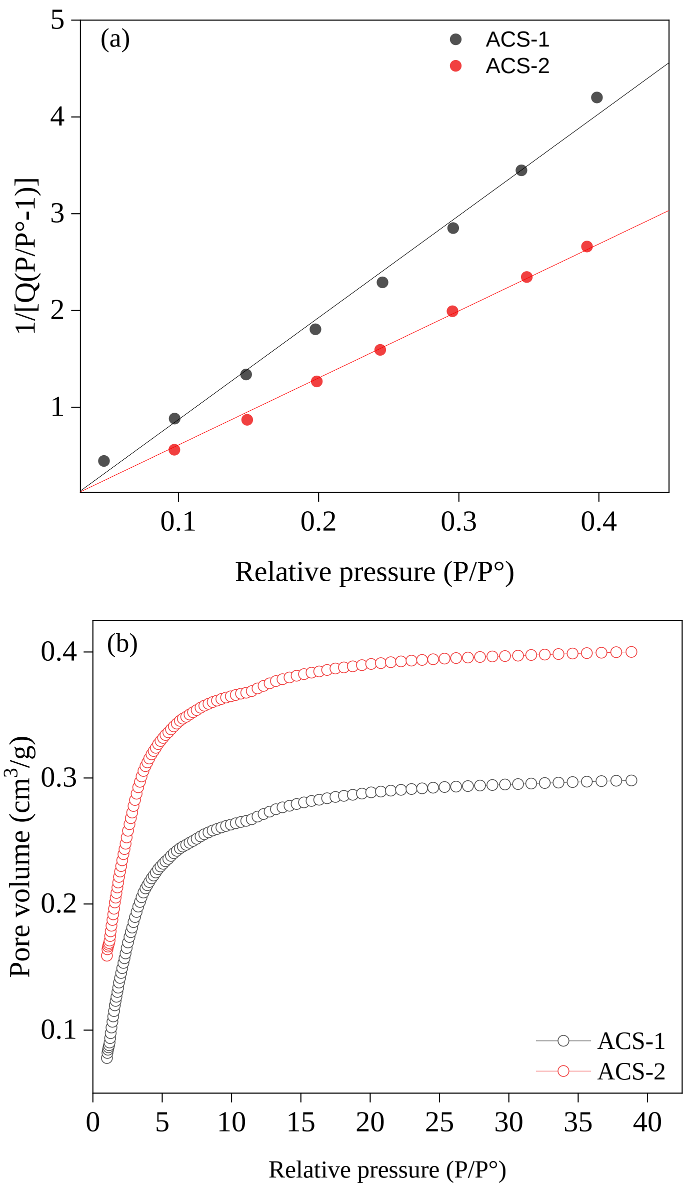
(a)
ACS-1
ACS-2
1/[Q(P/P°-1)]
Relative pressure (P/P°)
(b)
Pore volume (cm3/g)
ACS-1
ACS-2
Relative pressure (P/P°)

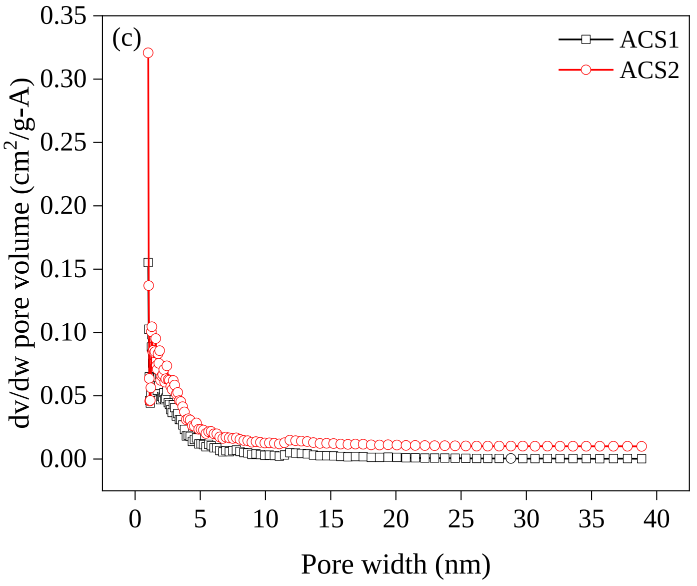

Figure S1. Surface area plot for (**a**) ACS1 and for ASC2, (**b**) BJH desorption P° distribution for ACS1 and for ACS2, and (**c**) differential pore volume plot for ACS1 and ACS2.

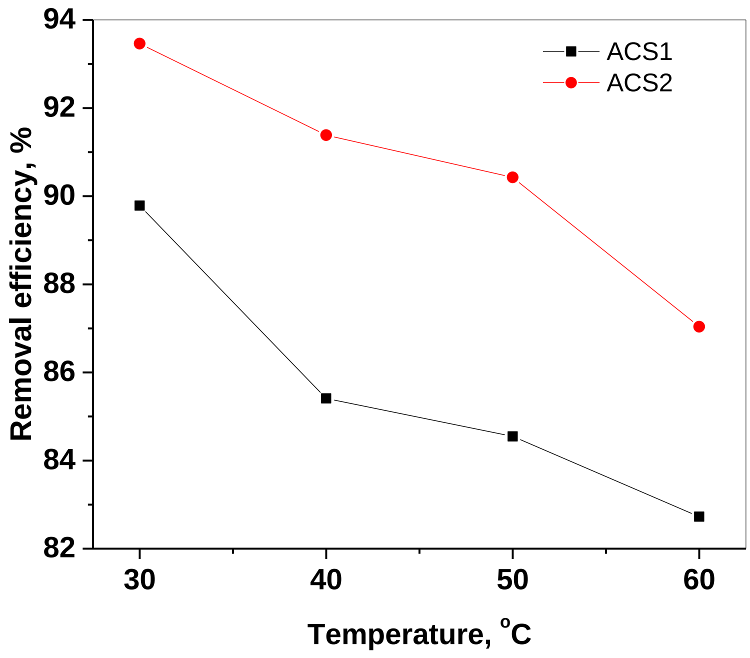

Figure S2. The impact of temperature variations on PR dye removal effectiveness using ACS1 and ACS2 at initial concentration; 100 mg, adsorbent dose; 40 mg and pH = 7.

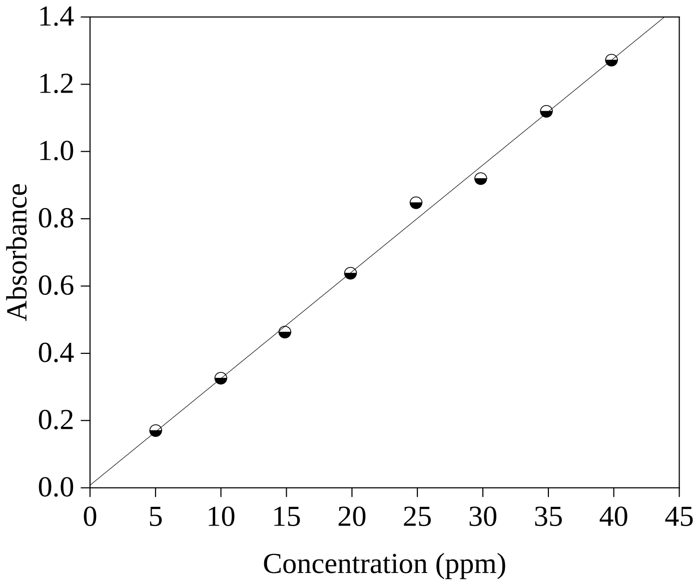

Figure S3. Calibration curve for determining the concentration of phenol red.
